# Cavalier King Charles Spaniels with Chiari-like malformation and Syringomyelia have increased variability of spatio-temporal gait characteristics

**DOI:** 10.1186/s12917-017-1077-5

**Published:** 2017-06-06

**Authors:** Emil Olsen, Emma Jane Suiter, Thilo Pfau, Imelda M McGonnell, Kaspar Matiasek, Anna Giejda, Holger Andreas Volk

**Affiliations:** 1 Department of Clinical Science and Services, The Royal Veterinary College, Hawkshead Lane, Hatfield, AL9 7TA UK; 2 Department of Clinical Sciences, Faculty of Veterinary Medicine, Cornell University, 930 Campus Road, Ithaca, N.Y. 14850 USA; 3 Department of Comparative Biomedical Sciences, The Royal Veterinary College, London, England UK; 4 Section of Clinical & Comparative Neuropathology, Centre for Clinical Veterinary Medicine, Ludwig Maximilians University, Veterinarstr, 13, D-80539 Munich, Germany

## Abstract

**Background:**

Chiari-like malformation in the Cavalier King Charles Spaniel is a herniation of the cerebellum and brainstem into or through the foramen magnum. This condition predisposes to Syringomyelia; fluid filled syrinxes within the spinal cord. The resulting pathology in spinal cord and cerebellum create neuropathic pain and changes in gait. This study aims to quantify the changes in gait for Cavalier King Charles Spaniel with Chiari-like malformation and Syringomyelia.

**Methods:**

We compared Cavalier King Charles Spaniel with Chiari-like malformation with (*n* = 9) and without (*n* = 8) Syringomyelia to Border Terriers (*n* = 8). Two video cameras and manual tracking was used to quantify gait parameters.

**Results and conclusions:**

We found a significant increase in coefficient of variation for the spatio-temporal characteristics and ipsilateral distance between paws and a wider base of support in the thoracic limbs but not in the pelvic limbs for Cavalier King Charles Spaniels compared with the border terrier.

**Electronic supplementary material:**

The online version of this article (doi:10.1186/s12917-017-1077-5) contains supplementary material, which is available to authorized users.

## Background

The Cavalier King Charles Spaniel (CKCS) is an example of a dog selectively bred for distinct conformational traits. The breed shows a high degree of juvenile behaviour and brachycephalic traits associated with a juvenile morphology (paedomorphic) when compared with the wolf and other companion dog breeds [1]. This selection for paedomorphism may have resulted in a specific morphology of the skull and brain, leading to a hereditary pathological mismatch between skull volume and brain volume, with a resulting herniation of part of the cerebellum and brainstem into or through the foramen magnum, a condition called Chiari-like Malformation (CM). Chiari-like malformation is linked to Syringomyelia (SM) which causes neuropathic pain [2]. CM is estimated to afflict up to 92% of the CKCS population [2]. It is believed that more than half of CKCS with CM will develop fluid-filled cavities within the spinal cord (SM) later in life and are affected with varying degrees of clinical signs. In fact, 25% of dogs with CM had SM at the age of 12 months increasing to 70% at 6 years of age [3]. In addition, the CKCS, when compared with other dogs, has a relatively larger cerebellum [2], which may predispose them to cerebellar dysfunction. The clinical signs of CM and SM include neck and head pain, cervical scoliosis, thoracic and pelvic limb ataxia (incoordination), thoracic limb paresis and neuropathic pain [4].

Coordination has been defined as “an ability to maintain a context-dependent and phase-dependent cyclical relationship between different body segments or joints in both spatial and temporal domains” [5]. Coordination of gait involves a complex interaction between proprioceptive receptors, stretch receptors in muscles, spinal cord, cerebellum, motor cortex and vision. If either of these pathways are diseased, such as in CM and SM in the CKCS, changes in the variation of gait are expected.

The cerebellum is known to adjust and control balance and locomotion, incorporating autonomous sensorimotor feedback from the spinal cord and feed-forward from the cerebral cortex and other primary and secondary motor centres of the brain [6]. The cerebellum also aids stabilisation of motor control [7] and inter-limb coordination [8]. Cerebellar ataxia is clinically characterised by dysmetria most commonly with hypermetria. Objective human gait analysis of patients with cerebellar ataxia show a) slower walk, prolonged duration of stance phase and increased variation of spatio-temporal characteristics and b) a wide based stance [9–11]. This increased variation of spatio-temporal characteristics is similar to that found in cats with experimentally induced spinal cord injury [12] and dogs with cervical spondylotic myelopathy [13]. We therefore hypothesised that CKCS diagnosed with CM or CM with SM, would have significantly increased a) variation of the spatio-temporal gait characteristics (stride length and duration of step cycle and stance) and b) wider base of support with an increased distance between pelvic and thoracic paws during walk. Because so many CKCS are affected and therefore cannot serve as controls and we therefore had to choose a control breed of similar weight and frame such as the Border Terriers, with no genetic predisposition for cerebellar or spinal cord disease. It should be mentioned that Border Terriers have been found to have paroxysmal movement disorders [14], and some puppies have been reported to have a leukoencephalomyelopathy [15] as well as a hypomyelination syndrome [16] where the latter two syndromes are present in young puppies.

## Methods

### Animals and study groups

Dogs were recruited into three groups; CKCS with CM only (Group CM), CKCS with CM and SM (Group CMSM) and Border Terriers. CKCS were recruited to the study if they had a neurological examination and an MRI report showing CM (<1 year) or CM and SM and the owner would consent to the dog participating in the study. Border Terriers were chosen as control breed (Group Control) due to their similar height and conformation to CKCS as described by the UK Kennel Club Breed Standards. Border Terriers were recruited for the study if they visited the QMH for routine veterinary work or elective procedures or through members of staff. Both CKCS and Control groups were examined and excluded from the study if the gait assessment showed clinical signs of lameness or systemic disease. The control group dogs were excluded if the owners reported concern of neurologic disease or if the gait evaluation showed ataxia. Measurements of distance between the shoulder joints were obtained and showed no difference between the CKCS and the control breed. The study was preapproved by the ethics committee of the Royal Veterinary College, approval number URN 2012 1139. All owners volunteering their dogs to the study signed a consent form explaining the details of the study.

### Data acquisition

Motion capture was performed in the structure and motion lab at the Royal Veterinary College as well as at the homes of participating dog owners unable to travel to the college campus. The dogs were walked over-ground along an 8 m runway with black electrical tape marking up 0.5 × 0.5 m squares into a 0.5 × 5 m grid made up of black tape on a flat surface.

**Fig. 1:**
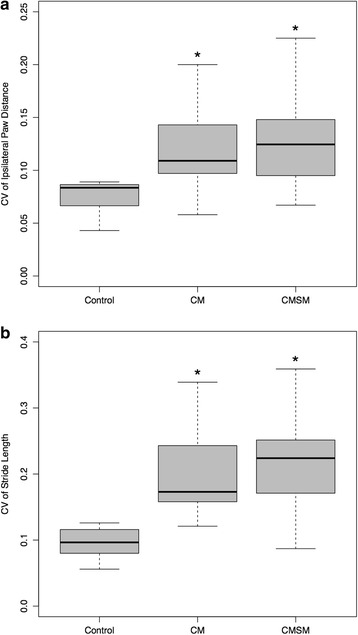
**a** Box plots showing the CV of ipsilateral paw distance between the control group (Control), CKCS with CM only (CM) and CKCS with CM and SM (CKCM). The *solid grey* of the boxes illustrate the 25–75% interquartile and the *black horizontal line* through each box is the median. The whiskers illustrate the minimum and maximum excluding outliers. Both CM and CMSM show increased CV and wider range of CV values when compared to the control group. **b** Box plots showing the CV of stride length between the control group (Control), CKCS with CM only (CM) and CKCS with CM and SM (CMSM). The *solid grey* of the boxes illustrate the 25–75% interquartile and the *black* *horizontal line* through each box is the median. The whiskers illustrate the minimum and maximum excluding outliers. Both CM and CMSM show increased CV and wider range of CV values when compared to the control group

Two digital stills set to film in full HD (1920 × 1080 pixels) and a frame-rate of 60 fps and were used in the study (EX-F1, Casio, Tokyo, Japan; 1-J1, Nikon, Tokyo, Japan). The cameras were set up more than 3 m away from the grid (Fig. 1) to view the entire grid and allow the zoom function to focus the grid into view. The Shutter speed was set at 1/1250 to reduce motion blur.

Dogs were walked at their preferred speed across the runway. The owners were asked to keep their dog within the grid and walk with steady pace. Trials were discarded if a dog changed gait or stopped during the trial. Trials were repeated until 50 accepted walking strides were collected. This data collection set-up is the same as reported by Suiter et al. [17].

### Data processing

A total of 6 gait parameters were chosen from the dogs during walk: Ipsilateral distance defined as the distance in cm between the thoracic limb paw placement and the ipsilateral pelvic limb paw placement. Stride length was defined as the distance in cm from where the middle toe of the paw of interest leaves the ground and where the same paw subsequently lands. Step cycle was defined as the time in seconds between the middle toe-off the ground and then the same toe of the paw being placed back on the ground. Stance time was defined as the time in seconds between the middle of the paw landing on the ground and then the same toe of the paw being lifted off the ground into the subsequent stride. The pelvic distance was defined as the distance in cm between placement of the left pelvic limb paw and the subsequent right pelvic limb paw placement. Thoracic distance was defined as the distance in cm between the placement of the left thoracic limb paw and the subsequent right thoracic limb paw placement.

The videos were processed with video analysis software (Quintic Biomechanics, v17, Quintic Consultance ltd, Coventry, UK). A frame-by-frame advancement was used in the software to calculate step cycle and stance time of each subject. In order to measure the other 4 parameters, the video analysis software (Quintic) was then used to obtain still images each time the subject placed a paw on the ground. The still images were saved as JPEG format. The still images were processed using an image processing and analysis tool (Image J, Open Source; *https://imagej.nih.gov/ij/download.html*). The image processing and analysis tool allowed for a scale to be set at 0.5 m matching the grid. Based on the reference frame ipsilateral distance, stride length, pelvic distance and thoracic distance were measured using the *‘straight line tool*’. To ensure measurements were from the same point on the foot for each individual, measuring started at the point in between the 2nd and 3rd digit of the foot when moving towards the camera. The middle of the plantar ball of the paw that is visible when the dog is moving away from the camera, and when in lateral view the measurement was taken from carpal joint down to the middle of the foot.

### Statistical analysis

The statistics were carried out using R version 2.15.3 [18] with the packages lattice for graphics [19] and pastecs [20] for descriptive statistics. To reduce the effect of the dogs walking at different speeds we calculated the coefficient of variation (CV) for each of the spatio temporal parameters. The larger the CV the more variable the stride parameters of the dog. Coefficient of variation (CV) was defined as standard deviation (SD) divided by the mean. The groups were compared using the package nlme [21] and a mixed model with the gait parameter or CV of gait parameters as outcome measure and random effects of dogs with fixed effects of group (control compared to CM or CMSM), height of the dog, age and disease (CM and CMSM) or no disease (Control). Because SM could influence gait patterns via dysfunction of the spinocerebellar tracts, CM and CMSM were also compared. The model was reduced using Akaike’s information Criterion. The significance level was set to *P* < 0.05.

## Results

A total of 25 dogs were recruited to the three groups with 9 dogs in group CM with a median age of 6 ranging from 3 years to 10 years, 8 dogs in group CMSM with a median age of 6.5 years ranging from 4 to 8 years. MRIs were performed on the CKCS dogs as they were enrolled in other studies or screened for breeding purposes. Additional file 1: Table S1 shows details of the clinical findings in the CKCS where none of the dogs in the CM group showed clinical signs and two of the CKCS in the SM group showed clinical signs of neuropathic pain with phantom scratching. Eight Border Terriers were recruited for the Control group with a median age of 5 years ranging from 8 months to 9 years. None of the CKCS or Border Terriers were on any medications.

The descriptive statistics are summarised in Table 1 and results of the mixed effect model comparisons between CM, CMSM and control groups are summarised in Table 2. Figure 1 illustrates the CV for each of the groups for ipsilateral distance between paws (Fig. 1a) and stride length (Fig. 1b). In the control group, 120–218 strides were accepted for the analysis of the spatiotemporal characteristics with an average of 27 stride cycles per dog ranging from 23 strides to 38 strides used with a mean stride length of 39 cm ± 10 cm. For the CKCS with CMSM, a total of 203 strides were included in the analysis with an average of 25 strides ranging from 19 to 40 steps with a mean stride length of 39 cm ± 22 cm. For the CKCS with CM, a total of 176 strides were included averaging 19 strides ranging from 18 to 21 strides per dog with a stride length of 43 cm ± 20 cm. From Table 2 it can be seen that only the distance between thoracic paws is significantly different when using the values for comparison of the control group with CKCS pooling both CM and CMSM groups (*p* = 0.02) and for the control group compared to CMSM (*p* = 0.01). When comparing the three groups and the CV of stride length, there is a significant difference between control group compared to either CKCS with CM (*p* = 0.003) or with CMSM (*p* = 0.001) as well as CM and CMSM pooled together (*p* < 0.001). When comparing the three groups and the CV of the ipsilateral paw distance, there is a significant difference between control group compared to either CKCS with CM (*p* = 0.04) or with CMSM (*p* = 0.02) as well as CM and CMSM pooled together (*p* = 0.01).

**Table 1:** Descriptive Statistics for spatio-temporal gait characteristics for CM, CMSM and control groups

Groups	Variable	Stride^e^ length	Ipsilateral paw distance^f^	Step cycle^g^	Stance time^h^	Pelvic distance^i^	Thoracic distance^j^
N steps (total)	-	597	531	382	548	548	545
Control (*n* = 8)	NumSteps^c^ (range/dog^d^)	218 (23–38)	185 (20–26)	120 (15)	187 (21–25)	189 (21–26)	188 (22–25)
CM^a^ (*n* = 9)	NumSteps^c^ (range/dog^d^)	176 (18–21)	172 (16–21)	140 (15–20)	176 (18–21)	176 (18–21)	176 (18–21)
CMSM^b^ (*n* = 8)	NumSteps^c^ (range/dog^d^)	203 (19–40)	174 (15–28)	122 (15–16)	185 (19–25)	183 (19–25)	181 (19–24)
Control (*n* = 8)	Mean (SD)	0.39 (0.10)	0.38 (0.08)	0.28 (0.17)	0.41 (0.54)	9.7 (0.32)	7.6 (0.45)
CM^1^ (*n* = 9)	Mean (SD)	0.43 (0.20)	0.44 (0.12)	0.28 (0.13)	0.27 (0.34)	10.8 (0.34)	9.3 (0.40)
CMSM^b^ (*n* = 8)	Mean (SD)	0.39 (0.22)	0.38 (0.13)	0.25 (0.18)	0.38 (0.65)	9.5 (0.27)	10.7 (0.34)
Control (*n* = 8)	CV	0.10	0.08	0.17	0.54	0.32	0.45
CM^a^ (*n* = 9)	CV	0.20	0.12	0.13	0.34	0.34	0.40
CMSM^b^ (*n* = 8)	CV	0.22	0.13	0.18	0.65	0.27	0.34

^a^ *CM* Chiari like malformation, ^b^ *CMSM* Chiari like malformation and syringomyelia, ^c^ *NumSteps* Total number of steps for all in the group ^d^ *Range* Ran2ge of steps from lowest per dog to highest per dog. ^e^ *Stride length* (*m*), Fore-aft distance (m) travelled by the paw during a stride, ^f^ *Ipsilateral paw distance* (*m*) Fore-aft distance between ground contact location from thoracic limb paw to pelvic limb paw, ^g^ *Step Cycle* Time in seconds from paw contact to the next paw contact on the same limb, ^h^ *Stance time* Time in seconds from paw contact to the paw is lifted from the ground, ^i^ *Pelvic distance* (*cm*), Latero-medial distance between contact location of the left pelvic limb paw to the right pelvic limb paw, ^j^ *Thoracic distance* (*cm*), Latero-medial distance between contact location of the left thoracic limb paw to the right thoracic limb paw

**Table 2:** Results of the mixed effect model comparisons between CM, CMSM and control groups

Comparison of Groups	Variable	Stride^e^ length	Ipsilateral paw distance^f^	Step cycle^g^	Stance time^h^	Pelvic distance^i^	Thoracic distance^j^
Control vs. CM^a^ & CMSM^b^	Value^c^	0.17	0.42	0.42	0.08	0.71	0.02^k^
Control vs CM^a^	Value	0.06	0.12	0.85	0.02^k^	0.48	0.11
Control vs CMSM^b^	Value	0.63	0.84	0.20	0.50	0.92	0.01^k^
Control vs. CM^a^ & CMSM^b^	CV^d^	<0.001^k^	0.01^k^	0.41	0.69	0.86	0.10
Control vs CM^a^	CV	0.003^k^	0.04^k^	0.05	0.22	0.67	0.33
Control vs CMSM^b^	CV	0.001^k^	0.02^k^	0.60	0.68	0.42	0.06

^a^ *CM* Chiari like malformation, ^b^ *CMSM* Chiari like malformation and syringomyelia, ^c^The actual values compared between groups, ^d^ *CV* Coefficient of Variation, ^e^ *Stride length* (*m*), Fore-aft distance (m) travelled by the paw during a stride, ^f^ *Ipsilateral paw distance* (*m*) Fore-aft distance between ground contact location from thoracic limb paw to pelvic limb paw, ^g^ *Step Cycle*, Time in seconds from paw contact to the next paw contact on the same limb; ^*h*^ *Stance time* Time in seconds from paw contact to the paw is lifted from the ground, ^i^ *Pelvic distance* (*cm*) Latero-medial distance between contact location of the left pelvic limb paw to the right pelvic limb paw, ^j^ *Thoracic distance* (*cm*): Latero-medial distance between contact location of the left thoracic limb paw to the right thoracic limb paw. ^k^Significant difference between groups

## Discussion

Firstly, we demonstrate increased variability through a significant increase in CV for the spatio-temporal characteristics for the CM group and the CMSM group compared with the control breed (Table 2 - and illustrated in Fig. 1). In particular, we find an increased CV of the ipsilateral distance between paws and length of the stride for both the CM and CMSM groups. Secondly, we demonstrate that CKCS in group CMSM, compared to the control group, have a significantly wider base of support in the thoracic limbs but not in the pelvic limbs (Table 2).

Motor control theories such as the uncontrolled manifold hypothesis and synergies are the current prevailing theories for how quadrupeds and bipeds maintain a tight control of coordination. The uncontrolled manifold theory suggests that the extremities and head aim to keep the centre of mass (CoM) as stable as possible by varying the trajectory of the extremities [22]. In a recent study of able-bodied subject and stroke patients Papi et al. [23] investigated the uncontrolled manifold method for both CoM and extremities and found less variation of extremity kinematic trajectory over time after the stroke with and without an orthoses. The decreasing variability of CoM and extremities over time in the stroke subject suggest a relearning of gait. It is not known how different components of the peripheral and central nervous system contributes to learning of gait but it is known that the cerebellum plays a central role in fine-tuning of gait and early learning of gait and other motor tasks [6]. Cerebellar deficits or cranial cervical myelopathies are therefore obvious possible explanations for the increased variation of spatio-temporal characteristics in the CKCS with CM or CMSM in our study. Dogs with spinal cord injuries have impaired coordination between thoracic and pelvic limbs [24]. There may be breed differences resulting in small variations of stability of distal limb spatio-temporal characteristics however these are not thoroughly described in the literature.

Children and adult humans with cerebellar ataxia and normal children show increased gait variability [11, 25–27]. Increased step width and variability of gait parameters could therefore be a primitive stabilising mechanism for both spinal cord and cerebellum. When compared to the control group, dogs with CM and CMSM exhibited a wider distance between thoracic paws. Similarly dogs with cervical spondylomyelopathy have increased distance between their thoracic but not pelvic limbs [13] and thus a wider base of support using the thoracic limbs may be a stabilising mechanism adapted for both cerebellar disease and spinal cord disease however breed effects cannot be ruled out. Measurements of distance between the shoulder joints were obtained and there was no difference between the CKCS and the control group, which confirmed that this observation is due to the CM and CMSM groups adopting a wider base of support rather than a result of differences in breed conformation.

The increased variability of ipsilateral distance between paws and stride length in both the CM and CMSM groups is similar to that documented in humans with cerebellar ataxia [9] as well as dogs with spinal cord injury [28, 29]. Furthermore, the increased variability is suggestive of an incoordination between pelvic and thoracic limbs with hypermetria, an abnormality of gait often associated with dysfunction of the cerebellum [11] or cranial spinal cord injury [13, 24]. The increased variation in distance between ipsilateral paws at the time of ground contact for the CM group and the CMSM group is similar to that reported in ataxic dogs with thoracolumbar spinal cord disease during walk on a treadmill [30]. The increased variability of step cycle duration for the CM group when compared to the control group is compatible with findings in dogs with spinal cord injury [31]. In our study we could not demonstrate an effect of SM alone on gait parameters, but there was an effect of the changes associated with CM which could suggest an involvement of the cerebellum in CM and fine-tuning of gait. The lack of differences between the CM and CMSM groups may be associated with breed specific differences or disease affection of the spinocerebellar tracts however greater numbers are necessary to elucidate the details of subtle gait changes associated with neuro-localisations.

The human preference for juvenile traits in the CKCS pedigree has had a significant impact on behaviour [1] where the CKCS has a reduced repertoire of normal adult behaviour such as absence of submissive signals and reduced threat behaviour such as baring teeth [1, 32]. We know that the CKCS has cerebellar pathology and syringomyelia [2] and with our demonstration of altered gait, it could be argued that we appear to maintain the dogs in a “puppy like state” throughout their lives.

### Study limitations

We have not investigated the spatio-temporal characteristics across multiple breeds as this is necessary to fully support the argument of cerebellar ataxia in CKCS with CM and CMSM and further studies should be performed of the breed to breed variations and effect of age and cerebellar disease on gait in dogs. We also recognise there is a correlation between the size of a dorsal syrinx and clinical signs of neuropathic pain (Rusbridge et al. [32]) and this may affect spatiotemporal gait characteristics.

Further the lack of control for gait speed when the dogs are walking along the runway could contribute to the stride-to-stride variation however this effect should not have a systematic bias between the groups. Future studies should include a neurologic examination of control dogs to improve the comparability.

## Conclusion

We conclude that Cavalier King Charles Spaniels with CM and CMSM have an increased variation of the ipsilateral distance between paws and length of the stride and a significantly wider base of support in the thoracic limbs but not in the pelvic limbs compared to Border Terriers. This could be an effect of cerebellar disease or cranial spinal cord disease however further breed-to-breed gait studies and objective quantification of hypermetria are necessary to further this conclusion. The increased spatial variation from CM and CMSM compared to control dogs could be used in rehabilitation and tracking of gait over time as well as monitoring for effect of treatment on gait changes and coordination.

## Additional files

Additional file 1: Table S1. Table showing all information of the dogs allocated to each of the three groups used in the proposed study. Grading is used to define the presence and stage of syringomyelia. (DOCX 14 kb)

Additional file 2: Figure S1. Representation of the camera setup and paw placements within the 0.5 × m grid. Two cameras are placed at the front and perpendicular to the grid, allowing video capture of both the forward and away movement as well as the lateral movement of the dog. Freeze frames were taken as each paw was placed flat on the ground and could then be used to calculate the parameters described. This figure displays how the pelvic distance is calculated. First the right pelvic paw was measured from the centre of the paw to the external grid line (blue line). The same was measured from the left pelvic paw to the same external grid line (green line). The two distances were then subtracted to result in the distance between the two pelvic paws (red line). (JPG 37 kb)

## Abbreviations

CKCS: Cavalier King Charles Spaniel
CM: Chiari-like Malformation
CMSM: Chari-like malformation and syringomyelia
CV: Coefficient of Variation
SD: Standard Deviation
SM: Syringomyelia
