# Supplementary material for: Cavalier King Charles Spaniels with Chiari-like malformation and Syringomyelia have increased variability of spatio-temporal gait characteristics
Source: BMC Vet Res. 2017 Jun 6;13:159. doi: 10.1186/s12917-017-1077-5 (PMC5461676; doi:10.1186/s12917-017-1077-5)
Supplement: Supplementary file 1 — Table showing all information of the dogs allocated to each of the three groups used in the proposed study. Grading is used to define the presence and stage of syringomyelia. (DOCX 14 kb) [file 12917_2017_1077_MOESM1_ESM.docx]

| **Groups** | **Variable** | **Stride^5^ length** | **Ipsilateral paw distance^6^** | **Step cycle^7^** | **Stance time^8^** | **Pelvic distance^9^** | **Thoracic distance^10^** |
| --- | --- | --- | --- | --- | --- | --- | --- |
| **N steps (total)** | - | 597 | 531 | 382 | 548 | 548 | 545 |
| **Control (n = 8)** | NumSteps^3^ (range/dog^4^) | 218 (23-38) | 185 (20-26) | 120 (15) | 187 (21-25) | 189 (21-26) | 188 (22-25) |
| **CM^1^ (n = 9)** | NumSteps^3^ (range/dog ^4^) | 176 (18-21) | 172 (16-21) | 140 (15-20) | 176 (18-21) | 176 (18-21) | 176 (18-21) |
| **CMSM^2^ (n = 8)** | NumSteps^3^ (range/dog ^4^) | 203 (19-40) | 174 (15-28) | 122 (15-16) | 185 (19-25) | 183 (19-25) | 181 (19-24) |
| **Control (n = 8)** | Mean (SD) | 0.39 (0.10) | 0.38 (0.08) | 0.28 (0.17) | 0.41 (0.54) | 9.7 (0.32) | 7.6 (0.45) |
| **CM^1^ (n = 9)** | Mean (SD) | 0.43 (0.20) | 0.44 (0.12) | 0.28 (0.13) | 0.27 (0.34) | 10.8 (0.34) | 9.3 (0.40) |
| **CMSM^2^ (n = 8)** | Mean (SD) | 0.39 (0.22) | 0.38 (0.13) | 0.25 (0.18) | 0.38 (0.65) | 9.5 (0.27) | 10.7 (0.34) |
| **Control (n = 8)** | CV | 0.10 | 0.08 | 0.17 | 0.54 | 0.32 | 0.45 |
| **CM^1^ (n = 9)** | CV | 0.20 | 0.12 | 0.13 | 0.34 | 0.34 | 0.40 |
| **CMSM^2^ (n = 8)** | CV | 0.22 | 0.13 | 0.18 | 0.65 | 0.27 | 0.34 |
| 1) CM: Chiari like malformation. 2) CMSM: Chiari like malformation and syringomyelia; 3) NumSteps: Total number of steps for all in the group 4) Range: Ran2ge of steps from lowest per dog to highest per dog. 5) Stride length (m): Fore-aft distance (m) travelled by the paw during a stride; 6) Ipsilateral paw distance (m): Fore-aft distance between ground contact location from thoracic limb paw to pelvic limb paw; 7) Step Cycle: Time in seconds from paw contact to the next paw contact on the same limb; 8) Stance time: Time in seconds from paw contact to the paw is lifted from the ground; 9) Pelvic distance (cm): Latero-medial distance between contact location of the left pelvic limb paw to the right pelvic limb paw; 10) Thoracic distance (cm): Latero-medial distance between contact location of the left thoracic limb paw to the right thoracic limb paw. | | | | | | | |

| **Comparison of Groups** | **Variable** | **Stride^5^ length** | **Ipsilateral paw distance^6^** | **Step cycle^7^** | **Stance time^8^** | **Pelvic distance^9^** | **Thoracic distance^10^** |
| --- | --- | --- | --- | --- | --- | --- | --- |
| **Control vs. CM^1^ & CMSM^2^** | Value^3^ | 0.17 | 0.42 | 0.42 | 0.08 | 0.71 | 0.02* |
| **Control vs CM^1^** | Value | 0.06 | 0.12 | 0.85 | 0.02* | 0.48 | 0.11 |
| **Control vs CMSM^2^** | Value | 0.63 | 0.84 | 0.20 | 0.50 | 0.92 | 0.01* |
| **Control vs. CM^1^ & CMSM^2^** | CV^4^ | < 0.001* | 0.01* | 0.41 | 0.69 | 0.86 | 0.10 |
| **Control vs CM^1^** | CV | 0.003* | 0.04* | 0.05 | 0.22 | 0.67 | 0.33 |
| **Control vs CMSM^2^** | CV | 0.001* | 0.02* | 0.60 | 0.68 | 0.42 | 0.06 |
| 1) CM: Chiari like malformation. 2) CMSM: Chiari like malformation and syringomyelia; 3) The actual values compared between groups; 4) CV: Coefficient of Variation; 5) Stride length (m): Fore-aft distance (m) travelled by the paw during a stride; 6) Ipsilateral paw distance (m): Fore-aft distance between ground contact location from thoracic limb paw to pelvic limb paw; 7) Step Cycle: Time in seconds from paw contact to the next paw contact on the same limb; 8) Stance time: Time in seconds from paw contact to the paw is lifted from the ground; 9) Pelvic distance (cm): Latero-medial distance between contact location of the left pelvic limb paw to the right pelvic limb paw; 10) Thoracic distance (cm): Latero-medial distance between contact location of the left thoracic limb paw to the right thoracic limb paw. *: Significant difference between groups | | | | | | | |

**Table S1**: Table showing all information of the dogs allocated to each of the three groups used in the proposed study. Grading is used to define the presence and stage of syringomyelia.

|  | **Dog Number** | **Gender** | **Age** | **Grade** |
| --- | --- | --- | --- | --- |
| Group 1  Control | 1 | Female | 8 months | NA |
|  | 2 | Male | 7 Years | NA |
|  | 3 | Female | 5 Years | NA |
|  | 4 | Male | 9 Years | NA |
|  | 5 | Male | 5 Years | NA |
|  | 6 | Male | 4 Years | NA |
|  | 7 | Male | 6 Years | NA |
|  | 8 | Female | 2 Years | NA |
| Group 2  CKCS CM | 1 | Male | 6 Years | A |
|  | 2 | Male | 7 Years | A |
|  | 3 | Female | 6 Years | A |
|  | 4 | Male | 3 Years | C |
|  | 5 | Male | 3 Years | C |
|  | 6 | Female | 6 Years | A |
|  | 7 | Male | 3 Years | C |
|  | 8 | Female | 7 Years | C |
|  | 9 | Male | 10 Years | C |
| Group 3  CKCS CMSM | 1 | Female | 7 Years | D |
|  | 2 | Female | 8 Years | D |
|  | 3 | Female | 4 Years | D |
|  | 4 | Female | 8 Years | F |
|  | 5 | Male | 8 Years | F |
|  | 6 | Female | 6 Years | D |
|  | 7 | Female | 4 Years | D |
|  | 8 | Female | 5 Years | D |
| Grade key: A; Over 2.5 years of age, absent of syrinxes or less than 2mm central canal dilation. C; Under 2.5 years with no syrinxes. D; Over 2.5 years, SM is present but animal is asymptomatic. F; At any age SM is present and the animal is symptomatic. CKCS: Cavalier King Charles Spaniel, CM: Chiari-like malformation, SM: Syringomyelia. The ages did not differ significantly between the groups according to a Kruskal-Wallis test (p<0.05). CV: Coefficient of Variation, mean calculated across individual dog CV. | | | | |
