# Supplementary figures and images for: Cavalier King Charles Spaniels with Chiari-like malformation and Syringomyelia have increased variability of spatio-temporal gait characteristics
Source: BMC Vet Res. 2017 Jun 6;13:159. doi: 10.1186/s12917-017-1077-5 (PMC5461676; doi:10.1186/s12917-017-1077-5)

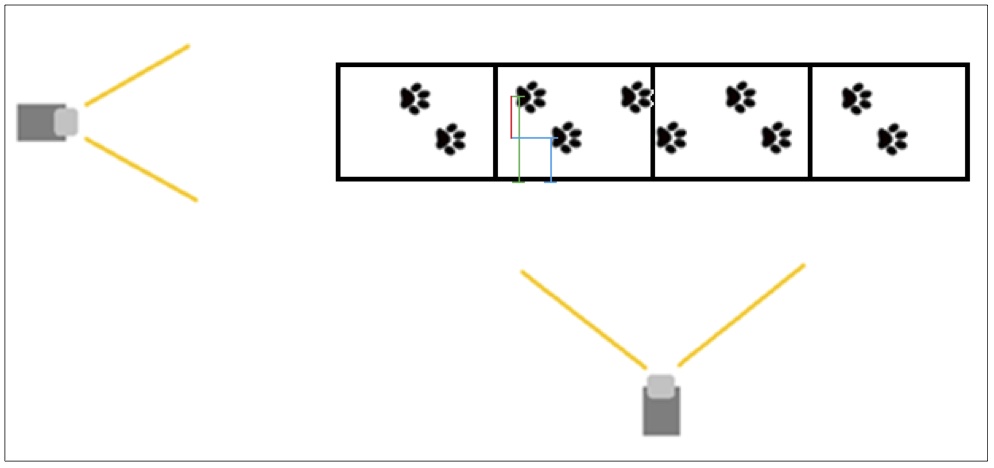

Supplement: Supplementary file 2 — Representation of the camera setup and paw placements within the 0.5 × m grid. Two cameras are placed at the front and perpendicular to the grid, allowing video capture of both the forward and away movement as well as the lateral movement of the dog. Freeze frames were taken as each paw was placed flat on the ground and could then be used to calculate the parameters described. This figure displays how the pelvic distance is calculated. First the right pelvic paw was measured from the centre of the paw to the external grid line (blue line). The same was measured from the left pelvic paw to the same external grid line (green line). The two distances were then subtracted to result in the distance between the two pelvic paws (red line). (JPG 37 kb) [file 12917_2017_1077_MOESM2_ESM.jpg]
